# Tick-Borne Pathogens in *Dermacentor reticulatus* Ticks from Bosnia and Herzegovina

**DOI:** 10.3390/pathogens13050421

**Published:** 2024-05-16

**Authors:** Teufik Goletić, Darinka Klarić Soldo, Naida Kapo, Šejla Goletić, Amira Koro-Spahić, Amra Alispahić, Adis Softić, Vedad Škapur, Jasmin Omeragić

**Affiliations:** 1 University of Sarajevo—Veterinary Faculty, 71000 Sarajevo, Bosnia and Herzegovina; darinka.klaric.soldo@vfs.unsa.ba (D.K.S.); naida.kapo@vfs.unsa.ba (N.K.); sejla.goletic@vfs.unsa.ba (Š.G.); amira.koro@vfs.unsa.ba (A.K.-S.); amra.alispahic@vfs.unsa.ba (A.A.); adis.softic@vfs.unsa.ba (A.S.); jasmin.omeragic@vfs.unsa.ba (J.O.); 2 University of Sarajevo—Faculty of Agriculture and Food Science, 71000 Sarajevo, Bosnia and Herzegovina; v.skapur@ppf.unsa.ba

**Keywords:** *Dermacentor reticulatus*, tick-borne pathogens, *Babesia* spp., *Anaplasma* spp., *Rickettsia* spp., *Borrelia burgdorferi* s.l., Bosnia and Herzegovina

## Abstract

*Dermacentor (D.) reticulatus* ticks carry and transmit a wide range of pathogens to vertebrate hosts. Limited information is available about the existence of emerging tick-borne pathogens and the distribution of *D. reticulatus* in Bosnia and Herzegovina. The study aimed to investigate the occurrence and distribution of *D. reticulatus* and to detect the presence of *Anaplasma* spp., *Borrelia (B.) burgdorferi* s.l., *Rickettsia* spp., and *Babesia* spp. in samples originating from questing ticks and ticks collected from domestic animals in various regions of Bosnia and Herzegovina. A total of 402 collected *D. reticulatus* ticks were widely distributed throughout the country. Of the 41 pools consisting of 205 individual *D. reticulatus* ticks, 21 (51.2%) indicated the presence of *Rickettsia* spp., 17 (41.4%) of *Babesia* spp., 2 (4.8%) of *Anaplasma* spp., and 1 (2.4%) of *B. burgdorferi* s.l. after real-time PCR screening. Our study indicates that *D. reticulatus* has significantly expanded its distribution and host range in Bosnia and Herzegovina. Moreover, our results represent the first detection of *Babesia* spp. in *D. reticulatus* in Bosnia and Herzegovina. Given the demonstrated presence of emerging pathogens in questing and feeding ticks, there is an urge to establish a surveillance system for ticks and tick-borne pathogens in Bosnia and Herzegovina.

## 1. Introduction

Ticks have exhibited a notable surge in population density and geographical range expansion across Europe during the initial two decades of the 21st century [1]. This can be attributed to a myriad of factors, with climatic change standing out as the primary driver [1]. However, it is crucial to recognize the multifaceted nature of this phenomenon, which also encompasses ecological alterations, shifts in the habitat of tick hosts, host variations, and human-induced modifications such as land use changes [2,3,4]. The proliferation of ticks into regions previously considered free from their presence has consequentially led to an escalation of tick-borne pathogens that have become a significant global public health challenge in recent years [5,6].

*Dermacentor reticulatus* ranks as the second most prevalent tick species within the Western Palearctic, succeeding *Ixodes ricinus* [7]. Its documented range of expansion is notable across various European countries [8,9,10]. In Bosnia and Herzegovina, an inventory of tick species has revealed the existence of eleven ixodid tick species to date [11]. Despite the fact that *I. ricinus* maintains its dominance as the primary tick species within the country, recent findings have highlighted the remarkable expansion of *D. reticulatus* ticks. This expansion is underscored by a significant increase in occurrence, escalating from a mere 0.2% in 2011 [12] to a notable 3.8% in 2022 [11]. Correspondingly, Bosnia and Herzegovina exhibited similar climatological trends observed across Europe, indicating a perceptible shift in *D. reticulatus* distribution. Notably, there has been a transition from its stronghold in the southern regions of the country, typified by a Mediterranean climate, to more northern territories, characterized by moderate-continental and mountain climates.

*Dermacentor reticulatus* shows resilient biological characteristics to conditions imposed by the environment, a broad host range, and notable vector capacity and competence, attributes congruent with its extensive dissemination throughout Europe and its veterinary and public health importance [8]. Given the intermittent feeding behavior of male ticks, *D. reticulatus* serves as a crucial vector for various pathogenic agents, distinguishing it epidemiologically from *Ixodes* spp. [8]. Notably, *D. reticulatus* stands as the primary vector of *Babesia canis*, alongside several bacterial pathogens, including *Anaplasma phagocytophilum*, *A. marginale*, *Bartonella* spp., *Coxiella burnetii*, *Francisella* spp., and *Rickettsia raoultii*. Additionally, it serves as a vector for viral pathogens such as the Omsk hemorrhagic fever virus and tick-borne encephalitis virus [8,9,13,14].

Several records of *Borrelia burgdorferi* sensu lato (s.l.), *Anaplasma phagocytophilum*, as well as various *Rickettsia* species, including *R. monacensis*, *R. helvetica*, *R. raoultii*, and *R. slovaca*, alongside *Francisella tularensis* subsp. holartica, have been documented in Bosnia and Herzegovina [15,16]. Conversely, the incidence of clinical cases pertaining to Lyme borreliosis and tularemia remains limited [17,18,19]. Despite these findings, the scientific literature concerning the presence of emerging infectious agents in ticks in Bosnia and Herzegovina is relatively lacking.

This study aimed to investigate the occurrence and geographical distribution of *D. reticulatus* while concurrently identifying the presence of *Anaplasma* spp., *Babesia* spp., *Borrelia (B.) burgdorferi* s.l., and *Rickettsia* spp. within ticks sampled from domestic animals and vegetation across diverse regions of Bosnia and Herzegovina.

## 2. Materials and Methods

### 2.1. Study Area and Sample Collection

**Figure 1:**
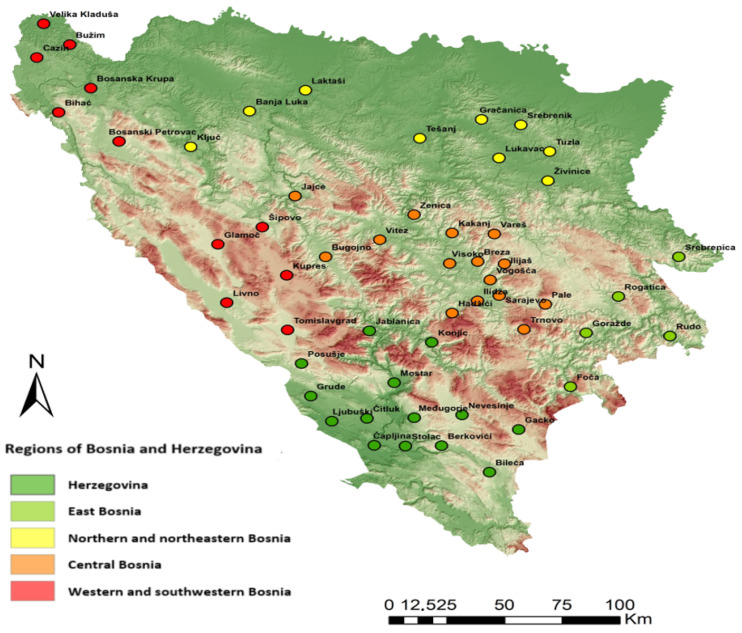
Map of Bosnia and Herzegovina with sampling sites included in the study. Colored circles represent the sampling sites (Software: QGIS ver. 3.28.8).

This study encompassed the entire state territory, with tick collections and sampling procedures meticulously tailored to reflect the geographical and bioclimatic attributes of the area. This study encompassed the entire state territory (Figure 1). Tick collections and sampling procedures were meticulously tailored to reflect the geographical and bioclimatic attributes of the study area and the distribution and prevalence of ticks within the territory as from previous studies.

The sampling of questing ticks involved techniques aimed at collecting ticks from environmental sources, e.g., vegetation, leaf litter, or other substrates, or ticks waiting to attach to a passing host. The sampling of questing ticks was performed by dragging a piece (1 × 1 square meter) of white fabric or flannel over the vegetation of leaf litter in accordance with standard protocols [20]. Ticks were then collected from the fabric using tweezers and placed in tubes until laboratory examination.

The selection of sampling sites was performed upon meticulous consideration of habitat characteristics, encompassing habitat type and vegetation density, alongside the adjacency to animal husbandry facilities, thereby targeting locales with higher potential for host abundance (Supplementary Table S1). Feeding ticks were collected directly from host animals, either with fine-tipped forceps or by gently dragging a cloth over the host’s fur. Another option involved a cloth that was gently dragged over the host’s fur, dislodging questing ticks onto the fabric for collection. The collection of feeding ticks was mainly performed by local veterinarians. From the total ticks collected (n = 7438), only *D. reticulatus* was selected for this study. Furthermore, these periods coincided with climatic conditions characterized by average monthly temperatures ranging from 13.0 and 20.0 °C, relative humidity exceeding 80%, and average monthly precipitation exceeding 120 mm. These conditions have been previously established as conducive to the prevalence and abundance of ticks throughout the territory of Bosnia and Herzegovina [12]. *D. reticulatus* was predominately sampled during two distinct periods: May to June and August to November, spanning seven consecutive years from 2017 to 2023. *D. reticulatus* collection was extended to the winter month of November, as previous observations reported its activity also during the winter season [11]. These sampling intervals were selected based on prior knowledge of the presence and distribution of ticks in Bosnia and Herzegovina [12]. After collection, *D. reticulatus* samples were transferred to plastic containers and transported to the University of Sarajevo—Veterinary Faculty. The sex and developmental stage of ticks were morphologically identified using a Zeiss Stemi 508 stereomicroscope (Zeiss, Oberkochen, Germany) with magnification 0.63–5.0×, employing established identification keys [21].

### 2.2. Tick Pooling Procedure

Following morphological identification, *D. reticulatus* was categorized and grouped based on developmental stage, sex, engorgement status, sampling details (including date and location), environmental characteristics (temperature and relative humidity), and host species from which they were collected. Upon collection, ticks were stratified based on their engorgement status, categorized as either engorged or non-engorged. Engorged ticks were identified by visual inspection of their size and appearance, indicative of blood ingestion. Non-engorged ticks were those lacking visible signs of blood feeding. A subset of the identified *D. reticulatus*, comprising 51% (205/402) of the total, was chosen for molecular analyses. Selection criteria for these ticks were based on the need to ensure adequate representation from all surveyed regions of Bosnia and Herzegovina, both urban and rural environments, as well as different types of host animals, including livestock and domestic pets. To prepare the pools, ticks were transferred from individual vials to sterile tubes using fine-tipped forceps, taking care to maintain the integrity of the specimens. The number of ticks in each pool was recorded, and the tubes were labeled accordingly to facilitate tracking and analysis. Each pooled sample comprised up to five adult ticks, with specific pooling protocols as follows: for instance, if ≥10 ticks of the same developmental stage were collected from a single host animal or herd, every second tick was selected for the pool. Likewise, if ≥20 ticks were present under similar conditions, every fourth tick was selected. The resulting pools were stored at −20 °C for further analysis.

### 2.3. Molecular Identification of Pathogens

The extraction of nucleic acids from pools was performed using a QIAamp DNA Mini kit (Qiagen, Hilden, Germany), according to the manufacturer’s instructions. Before DNA extraction, engorged ticks were carefully dissected, and each tick was cut in half using sterile scissors. The dissected tick halves were then individually homogenized in lysis buffer using a TissueLyser II (Qiagen, Hilden, Germany) to ensure efficient disruption of the tick tissues and facilitate DNA release. Homogenization was carried out at high speed to achieve thorough tissue disruption. Subsequently, DNA extraction was performed from the homogenized samples following the standard protocol provided by the manufacturer. The remaining halves of the dissected ticks were preserved by deep freezing as a backup for potential future analysis (e.g., identification of positive individual ticks) or other purposes. Subsequent DNA amplification procedures were executed utilizing the QuantiFast Pathogen PCR + IC Kit (Qiagen, Hilden, Germany) on the Mic qPCR Cycler platform (Bio Molecular Systems, Australia). The specific primers and probes, as well as pertinent information on the targeted pathogens, are outlined in Table 1. The multiplex PCR approach was implemented for selected pathogens, comprising two duplex PCRs targeting *Rickettsia* spp. and *Babesia* spp., as well as *Anaplasma* spp. and *B. burgodrferi* s.l. The identifications of *Rickettsia* spp., based on the amplification of a section of the gltA gene, *Babesia* spp., based on the amplification of the section of the 18S rRNA gene, *Borrelia burgdorferi* s.l., based on the amplification of the ospA gene, and *Anaplasma* spp., based on the amplification of Msp2 genes, were performed following previously published studies [22,23,24,25]. The cycling conditions used for both duplex PCRs were uniform, comprising an initial denaturation of 95 °C for 5 min, followed by 40 cycles of denaturation at 95 °C for 15 s, and annealing and elongation at 60 °C for 30 s. Fluorescence readings and graphical plotting were performed automatically at 60 °C after each extension/elongation phase, facilitated by the micPCR software ver. 2.8.13. (Bio Molecular Systems, Upper Coomera, Australia).

The prevalence of positive pooled tick samples was determined using a modified calculation method to account for the pooling of individual ticks. The prevalence was calculated by dividing the number of positive pools by the total number of pools tested. To adjust for pool size, the Clopper–Pearson method was employed. The prevalence calculations were conducted using Stata Statistical Software 15.1 (StataCorp LLC, College Station, TX, USA).

## 3. Results

A total of 402 *D. reticulatus* ticks (5.4%; 402/7438) were collected during the period 2017–2023. A total of 41 pools, including 205 individual *D. reticulatus* ticks, were meticulously prepared for subsequent analysis.

**Figure 2:**
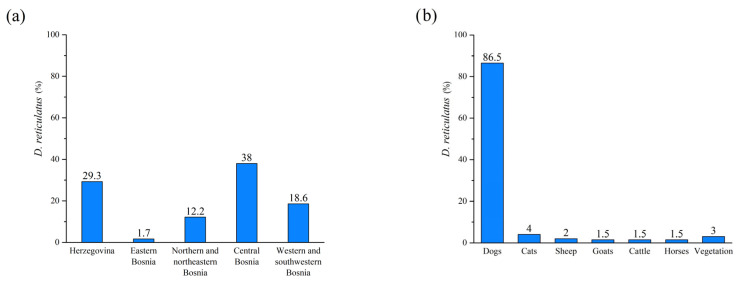
Distribution of *D. reticulatus* ticks in (**a**) geographical regions of Bosnia and Herzegovina and (**b**) source of collection.

**Figure 3:**
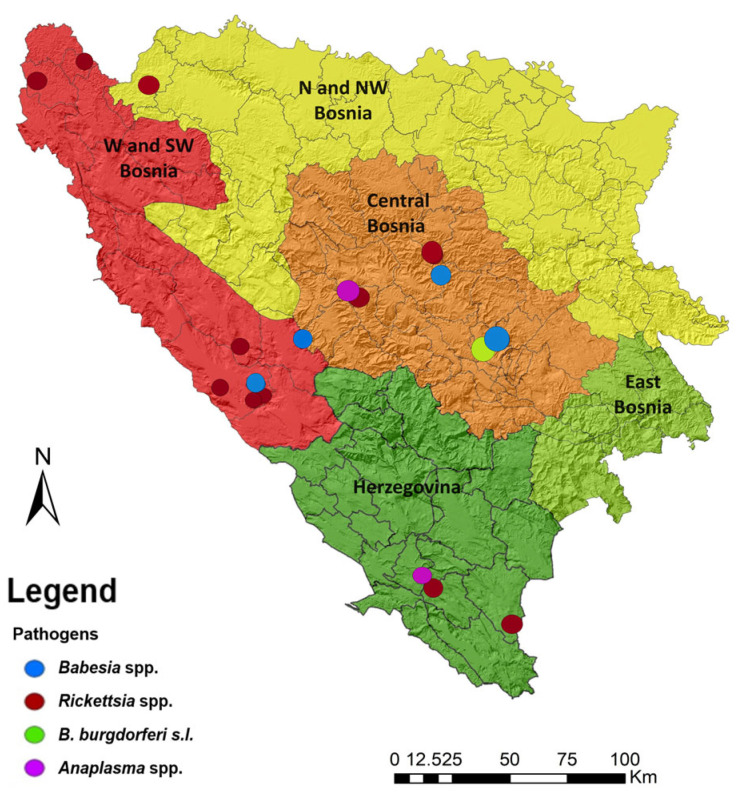
Distribution of tick-borne pathogens detected in different regions of Bosnia and Herzegovina.

The distribution pattern of *D. reticulatus* ticks across the study area and their associated host species is depicted in Figure 2. The distribution of tick-borne pathogens detected in *D. reticulatus* ticks in Bosnia and Herzegovina is depicted in Figure 3. It illustrates the cumulative distribution of tick-borne pathogens detected in *D. reticulatus* ticks across various regions of Bosnia and Herzegovina. Each color represents a different pathogen species.

**Figure 4:**
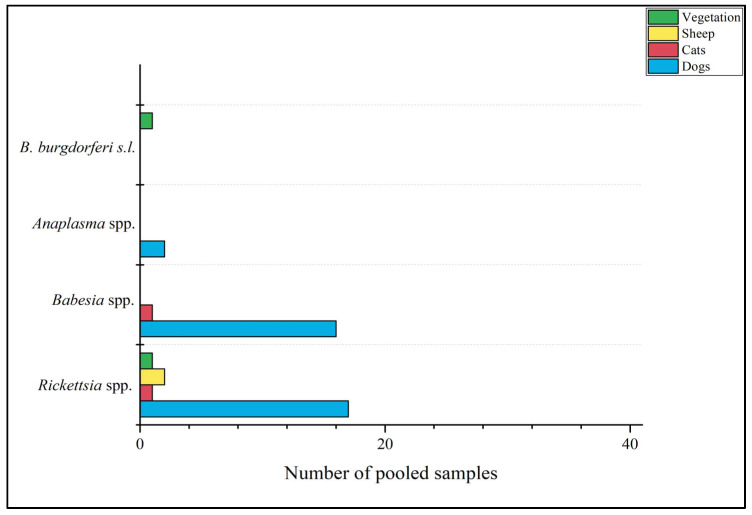
Positive *D. reticulatus* pools for at least one of the investigated pathogens distributed over the host type or vegetation in Bosnia and Herzegovina.

The findings reveal that 36 out of 41 *D. reticulatus* pools (87.8%) tested positive for at least one pathogen. Notably, the majority of pools (29 out of 41) exclusively consisted of female ticks, all of which (100%) were found to harbor at least one of the investigated pathogens. In contrast, a lower proportion of pools (7 out of 41) included only male ticks, with 58.3% of them testing positive for at least one pathogen. The distribution of positive pools, categorized by ticks’ sex and identified pathogens, is provided in Table 2. Furthermore, the spatial distribution of identified pathogens across the study regions is detailed in Table 3. Positive pools were obtained predominantly from dogs, but also cats, sheep, and questing ticks from vegetation were represented (Figure 4). However, specimens were also obtained from feline, ovine, equine, and bovine hosts, albeit at a slightly lower frequency.

An intriguing finding revealed that 14.6% (6 out of 41) of the pools exhibited positivity for at least two of the targeted pathogens. Specifically, mutual infection involving *Babesia* spp. and *Rickettsia* spp. was detected in four pools originating from dogs as well as one pool originating from sheep located in the western and southwestern Bosnia region. Upon further investigation, individual testing of ticks constituting these pools elucidated the presence of three ticks originating from two pooled dog samples, serving as carriers of both pathogens. In the Herzegovina region, another notable result arose when a pool collected from a dog showed positivity for *Rickettsia* spp. and *Anaplasma* spp. However, subsequent analysis at the individual level did not reveal co-infections.

## 4. Discussion

The overall share of *D. reticulatus* ticks among the collected tick specimens amounted to 5.4%. Our findings indicate a notable increase in the occurrence of *D. reticulatus* compared to prior studies conducted within Bosnia and Herzegovina [11,12]. Moreover, our sampling efforts extended into areas where *D. reticulatus* had not previously been documented, suggesting a notable shift in the distribution of this tick species and its habitat range, aligning with observations from earlier investigations [2,7]. Moreover, our findings offer an updated perspective on the scarcity of *D. reticulatus* ticks in the Balkan region, as suggested in a comprehensive review by Földvári et al. [8]. This expansion into new habitats may be attributed to the availability of uncultivated open environments in Bosnia and Herzegovina, but also across all of Europe. These habitats provide suitable conditions for tick survival because they are characterized by favorable microclimatic conditions and vegetation cover [26,27]. Additionally, our study revealed the occurrence of *D. reticulatus* in ruminants, increasing the host spectrum identified in our previous studies [11,12]. However, it is important to note that our study period spans from 2017 to 2023, after the timeframe covered in the publication by Földvári et al. [8]. Therefore, our results may offer an updated perspective on the distribution of *D. reticulatus* ticks in part of the Balkan region, i.e., Bosnia and Herzegovina.

**Figure 5:**
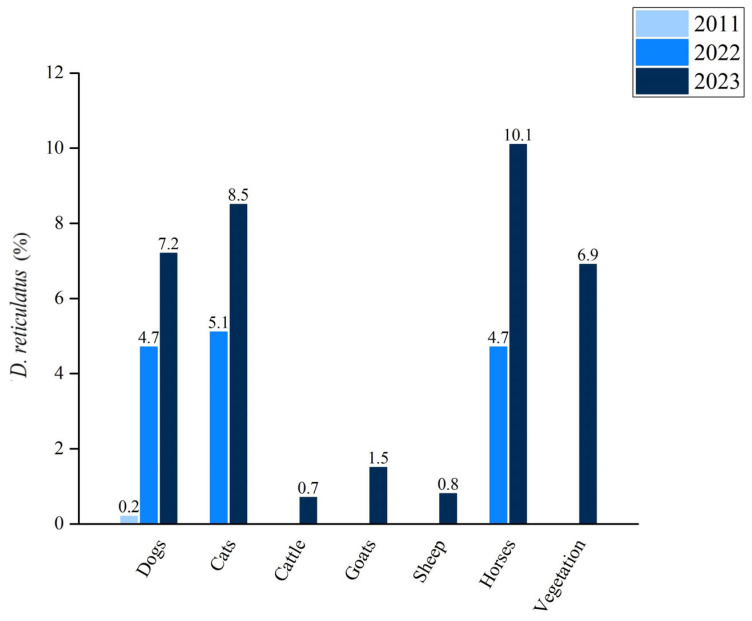
Percentage of *D. reticulatus* ticks collected from different animal hosts or questing from vegetation in 2011 [12], 2022 [11], and 2023 (this study) in Bosnia and Herzegovina.

The findings of our study revealed that *D. reticulatus* was predominantly collected from dogs, which is consistent with recent research findings [11]. Importantly, our study also observed a broader host range for *D. reticulatus*, with instances of their presence on cattle, goats, sheep, and vegetation, contrary to previous reports where this tick species was not documented on these animals or vegetation [11]. Furthermore, our results highlight variations in the prevalence of *D. reticulatus* among different host types and vegetation, as depicted in Figure 5. The expansion in the host range and distribution of these ticks might have substantial implications for tick-borne disease epidemiology and underscores the need for continued surveillance and research efforts to better understand the dynamics of tick populations and their associated pathogens.

Our study revealed that a considerable proportion of *D. reticulatus* pools sampled from hosts and vegetation across various regions, excluding eastern Bosnia, harbored at least one pathogen, with an overall prevalence of 87.8%.

The DNA of *Rickettsia* spp. was detected in 51.2% of *D. reticulatus* pools. *Rickettsia* spp. predominated in ticks collected from dogs, with 80.2% of positive pools originating from this host species. Additionally, *Rickettsia* spp. DNA was identified in one pool from cats in central Bosnia, as well as two pools from sheep in locations within Herzegovina and southwestern Bosnia. Prior studies conducted in Bosnia and Herzegovina identified *R. raoultii* and *R. slovaca* in *D. reticulatus* ticks. While our study did not specifically identify these species, our findings contribute to the body of knowledge regarding tick-borne pathogens in the region. Remarkably, a previous investigation compared the presence of both *Rickettsia* spp. in a subset of 54 *D. reticulatus* ticks collected from the vegetation [16]. Considering the evident increase in the distribution of *D. reticulatus* and its expanded habitat range, our results suggest the potential emergence of *Rickettsia* spp. as a new public health problem in Bosnia and Herzegovina. The detection of these pathogens in a substantial proportion of tick pools underscores the need for continued surveillance and research efforts to monitor and mitigate the risks associated with tick-borne diseases in Bosnia and Herzegovina.

In the present study, the detection of *Babesia* spp. in 41.4% of *D. reticulatus* pools represents a remarkable finding, marking the first report of *Babesia* spp. presence in *D. reticulatus* in Bosnia and Herzegovina. In previous investigations, the presence of *B. canis* was documented, with studies revealing its detection in 85% of dogs exhibiting clinical symptoms of the disease [28]. Moreover, the prevalence of *Babesia* spp. among dogs infested with ticks has been reported in 27.9% of asymptomatic and 43.2% of symptomatic dogs in Bosnia and Herzegovina [29]. Hodžić et al. [30] extended the understanding of *Babesia* spp. distribution by identifying the pathogen in red foxes (*Vulpes vulpes*), reporting that 0.8% of examined samples were infested with *B. canis* and 31.9% with *B. vulpes*. A distinct strain of *Babesia* spp., referred to as “Badger type A”, was isolated from a single sample obtained from a European wild cat (*Felis silvestris*) in Bosnia and Herzegovina [31]. Stevanović et al. [32] documented two cases of bovine babesiosis caused by *B. divergens* in central Bosnia and Herzegovina. We found *Babesia* spp. in a pool of female *D. reticulatus* ticks collected from a cat. While feline babesiosis is uncommon in Europe [33], our finding emphasizes the need for more research on the prevalence and impact of this parasite in cats in Bosnia and Herzegovina. Continued surveillance efforts are crucial to better understanding the distribution and significance of *Babesia* spp. in feline populations. However, it is important to note that the specific *Babesia* species was not identified, which limits our ability to draw definitive conclusions about feline babesiosis.

The presence of *B. burgdorferi* s.l. in *D. reticulatus* ticks provides additional evidence of its association with this tick species [34]. In northwestern Europe, *Borrelia afzelii*, *B. garinii*, *B. spielmanii*, and *B. miyamotoi* have been identified in *D. reticulatus* ticks collected from the vegetation, highlighting the presence of these pathogens in association with this tick species across the area [13]. While the detection of these pathogens in *D. reticulatus* ticks is noteworthy, it does not necessarily imply vector competence. Consistent with findings from previous investigations in northwestern Europe, our single positive pool for *B. burgdorferi* s.l. included ticks collected from vegetation in the central Bosnia region. The identification of this pathogen in questing *D. reticulatus* ticks raises concerns for at-risk human activities such as hunters, trekkers, foresters, pet owners, etc.

*Anaplasma* spp. was identified in two *D. reticulatus* pools (4.8%, 2/41) obtained from dogs in the central Bosnia region. Previous investigations targeting the presence of zoonotic bacteria within the tick population in Bosnia and Herzegovina reported the detection of *A. phagocytophilum* in 1.1% of examined female *D. reticulatus* ticks [16]. Notably, *A. phagocytophilum* and *A. marginale* were documented in infected *D. reticulatus* ticks sampled from the environment in northwestern Europe and Ukraine, although at a low prevalence, i.e., 0.28% and 0.1%, respectively [13,35]. Sprong et al. [13] could not confirm *A. phagocytophilum* using conventional PCR methods. These authors pointed out that it is important to note that molecular techniques alone do not provide conclusive evidence of the presence of this infectious agent in *D. reticulatus* ticks or its vector competence for *Anaplasma* spp. Therefore, further research is needed to elucidate the vector competence of *D. reticulatus* before the implications of these pathogen findings can be fully understood for the surveillance of vector-borne diseases.

In the western and southwestern regions of Bosnia, mutual infection with *Rickettsia* spp. and *Babesia* spp. was identified in five pools of *D. reticulatus* ticks, comprising four pools collected from dogs and one pool from sheep. Notably, three individual ticks were found to harbor both pathogens concurrently. Additionally, in the Herzegovina region, one pool collected from a dog exhibited mutual infection with *Rickettsia* spp. and *Anaplasma* spp. The detection of ticks harboring multiple pathogens suggests the potential for humans to acquire simultaneous infections as well. Co-infections pose a significant challenge in understanding the epidemiology of tick-borne diseases and complicate diagnosis in humans [36]. Given the fact that coinfections of individual ticks were observed in our study, our results call for attention to prospective public or animal health concerns in Bosnia and Herzegovina. Our study included an individual screening of ticks in cases where co-infection or mutual infections were suspected based on initial pool screening results; however, this approach may have limitations. The selective nature of individual screening may have resulted in underestimation or missed detection of certain pathogens, particularly those with low prevalence or those that may not have been identified during initial pool screening. Therefore, future studies should consider the feasibility of comprehensive individual screening of ticks to enhance the accuracy and robustness of pathogen detection.

## 5. Conclusions

The results of this study offer valuable insights into the emergence and spread of pathogens carried by *D. reticulatus* in Bosnia and Herzegovina. Our results shed light on the expanding distribution and widening of the host spectrum of *D. reticulatus*, contributing to a deeper understanding of the epidemiology of tick-borne diseases within the region. The increasing prevalence and geographical range of *D. reticulatus* underscore the need for enhanced surveillance and control measures specific to Bosnia and Herzegovina, but also in neighboring countries and regions where similar ecological conditions may support the establishment and spread of this tick species. The detection of emerging tick-borne pathogens in *D. reticulatus* raises concerns about the potential for spillover into human populations. Given the interconnected nature of ecosystems and the mobility of hosts and vectors, the threat posed by tick-borne diseases transcends national boundaries, highlighting the importance of regional and international cooperation in addressing this public health challenge. In light of these findings, there is a pressing need for collaborative efforts among researchers, public health authorities, and policymakers to develop and implement integrated strategies for the prevention, surveillance, and control of tick-borne diseases. This includes the establishment of comprehensive surveillance programs, the development of targeted interventions to reduce tick populations and mitigate human exposure, and the promotion of public awareness and education initiatives to empower individuals to protect themselves against tick bites and tick-borne infections within Bosnia and Herzegovina

## Figures and Tables

**Table 1:** Primers and probes used for molecular detection of bacterial and protozoan pathogens in *D. reticulatus* ticks (real-time PCR).

Primer (100 pmol/µL)	Oligonucleotide Sequence (5′–3′)	Final Conc./25 µL	Amplicon Size (bp)	Ref.
Rspp-F	GAGAGAAAATTATATCCAAATGTTGAT	450 nM	100	[20]
Rspp-R	AGGGTCTTCGTGCATTTCTT			
Rspp-P	[CY5]-CATTGTGCCATCCAGCCTACGGT-[BHQ2]	150 nM		
Bab-F	CAGCTTGACGGTAGGGTATTGG	1 µM	20	[21]
Bab-R	TCGAACCCTAATTCCCCGTTA			
Bab-P	[YAKYE]- CGAGGCAGCAACGG-[BHQ1]	500 nM		
Bbsl_ospA-F	AATATTTATTGGGAATAGGTCTAA	600 nM	59	[22]
Bbsl_ospA-R	CACCAGGCAAATCTACTGA			
Bbsl_ospA-P	[FAM]-TTAATAGCATGTAAGCAAAATGTTAGCA-[BHQ1]	100 nM		
Apmsp2-F:	ATGGAAGGTAGTGTTGGTTATGGTATT	900 nM	77	[23]
Apmsp2-R	TTGGTCTTGAAGCGCTCGTA			
Apmsp2-P	[CY5]-TGGTGCCAGGGTTGAGCTTGAGATTG-[BHQ2]	125 nM		

**Table 2:** Positive *D. reticulatus* pools according to pathogens distributed over the sex of ticks.

Sex	*Rickettsia* spp.	*Babesia* spp.	*Anaplasma* spp.	*Borrelia burgdorferi* s.l.	Positive Pools
Female (n = 29)	62.0% (18/29) CI *: 42.3–79.3	48.3% (14/29) CI: 29.5–67.5	3.4% (1/29) CI: 0.01–17.8	3.4% (1/29) CI: 0.01–17.8	100% (29/29)
Male (n = 12)	25.0% (3/12) CI: 5.5–57.2	25.0% (3/12) CI: 5.5–57.2	8.3% (1/12) CI: 0.2–38.5	-	58.3% (7/12)

* 95% Confidence interval.

**Table 3:** Positive *D. reticulatus* pools according to pathogens and geographical regions in Bosnia and Herzegovina.

Pathogens	W and SW Bosnia	Herzegovina	Central Bosnia	N and NW Bosnia	E Bosnia
*Rickettsia* spp.	28.6% (6/21) CI: 11.3–52.2	57.1% (12/21) CI: 34.0–78.2	4.7% (1/21) CI: 0.1–23.8	9.5%, (2/21) CI: 1.2–30.4	-
*Babesia* spp.	70.6%, (12/17) CI: 44.0–89.7	-	29.4%, (5/17) CI: 10.3–56.0	-	-
*Anaplasma* spp.	-	50.0%, (1/2) CI: 1.3–98.8	50.0%, (1/2) CI: 1.3–98.8	-	-
*B. burgdorferi* s.l.	-	-	100%, (1/1) CI: 2.5–100	-	-

## Data Availability

Data are contained within the article and Supplementary Materials.

## Supplementary Materials

The following supporting information can be downloaded at https://www.mdpi.com/article/10.3390/pathogens13050421/s1, Table S1: Meta data about sampling sites and methods, climatic condition and habitat in selected study areas of Bosnia and Herzegovina.
