# Supplementary material for: Tick-Borne Pathogens in Dermacentor reticulatus Ticks from Bosnia and Herzegovina
Source: Pathogens. 2024 May 16;13(5):421. doi: 10.3390/pathogens13050421 (PMC11123776; doi:10.3390/pathogens13050421)
Supplement: Supplementary file 1 [file pathogens-13-00421-s001.zip › pathogens-2907009-supplementary.pdf]

Supplementary Table 1. Meta data about **sampling sites and methods, climatic condition and habitat in selected study areas of Bosnia and Herzegovina**

Date	Municipality	Location	Adult F	Adult M	Total	Tick species	Sampling method	Host	Weather	T (°C)	Biotopes	Region B&H
10-03-23	Livno	Žabljak	1	1	2	D. reticulatus	manual collection	sheep	Sunny	(-6 plus minus 3)		W and SW Bosnia
10-03-23	Livno	Guber	3	2	5	D. reticulatus	manual collection	dog	Sunny	(-6 plus minus 3)		
19-04-23	Cazin	Krivaja	1	0	1	D. reticulatus	manual collection	dog	Sunny	5°C		
24-04-23	Kupres	Kupres	1	0	1	D. reticulatus	manual collection	sheep	Sunny	12°C		
24-04-23	Tomislavgrad	Tomislavgrad	3	0	3	D. reticulatus	manual collection	sheep	Sunny	13°C		
08-10-23	Bihać	Bihać	1	0	1	D. reticulatus	manual collection	dog	Sunny	22°C		
16-03-22	Bihać	Bihać	1	0	1	D. reticulatus	manual collection	dog	Sunny	14°C		
16-03-22	Cazin	Krivaja	0	1	1	D. reticulatus	manual collection	dog	Sunny	14°C		
16-03-22	Velika Kladuša	Velika Kladuša	1	0	1	D. reticulatus	manual collection	dog	Sunny	13-16°C		
23-09-22	Glamoč	Glamoč	1	0	1	D. reticulatus	manual collection	dog	Sunny	11°C		
06-02-21	Livno	Komorani	2	1	3	D. reticulatus	manual collection	dog	Cloudy	Unrecorded		
20-05-21	Livno	Lištani	2	0	2	D. reticulatus	manual collection	dog	Cloudy	13°C		
22-03-21	Bosanski Petrovac	Smoljana	2	2	4	D. reticulatus	manual collection	dog	Sunny	10°C		
20-05-21	Livno	Guber	2	1	3	D. reticulatus	manual collection	dog	Sunny	16°C		
14-04-21	Bosanski Petrovac	Bosanski Petrovac	1	1	2	D. reticulatus	manual collection	cattle	Cloudy	17°C		
20-05-21	Livno	Podkraj	2	0	2	D. reticulatus	manual collection	dog	Sunny	15°C		

04-09-21	Livno	Suhača	1	0	1	D. reticulatus	manual collection	dog	Sunny	15°C	
04-09-21	Livno	Suhača	2	0	2	D. reticulatus	manual collection	cat	Sunny	15°C	
04-09-21	Livno	Žabljak	1	1	2	D. reticulatus	manual collection	dog	Sunny	15°C	
28-02-21	Kupres	Kupres	2	0	2	D. reticulatus	manual collection	horse	Unrecorded	10°C	
12-03-21	Tomislavgrad	Tomislavgrad	3	1	4	D. reticulatus	manual collection	dog	Unrecorded	7°C	
22-03-21	Cazin	Klis	1	0	1	D. reticulatus	manual collection	dog	Unrecorded	16°C	
22-03-21	Bosanska Krupa	Bosanska Krupa	2	1	3	D. reticulatus	manual collection	goat	Unrecorded	16°C	
24-03-21	Bužim	Bužim	2	1	3	D. reticulatus	manual collection	dog	Unrecorded	16°C	
24-03-21	Bihać	Bihać	3	0	3	D. reticulatus	manual collection	dog	Unrecorded	16°C	
24-03-21	Glamoč	Glamoč	2	0	2	D. reticulatus	manual collection	dog	Unrecorded	17°C	
27-03-20	Cazin	Cazin	1	0	1	D. reticulatus	manual collection	dog	Unrecorded	10°C	
10-03-20	Livno	Žabljak	2	0	2	D. reticulatus	manual collection	dog	Sunny	Unrecorded	
23-10-20	Livno	Guber	2	0	2	D. reticulatus	manual collection	dog	Sunny	23°C	
23-10-20	Livno	Suhača	3	0	3	D. reticulatus	manual collection	dog	Cloudy	22°C	
23-10-20	Livno	Livno	1	0	1	D. reticulatus	manual collection	dog	Sunny	13°C	
23-10-20	Livno	Podhum	2	0	2	D. reticulatus	manual collection	dog	Cloudy	20°C	
08-04-18	Tomislavgrad	Tomislavgrad	1	0	1	D. reticulatus	manual collection	dog	Unrecorded	18°C	
05-05-18	Bužim	Bužim	1	0	1	D. reticulatus	manual collection	dog	Unrecorded	21°C	
18-05-18	Livno	Livno	1	0	1	D. reticulatus	dragging	vegetation	Unrecorded	23°C	meadow

19-10-18	Bužim	Bužim	1	0	1	D. reticulatus	manual collection	dog	Unrecorded	18°C		
14-05-18	Bihać	Bihać	1	0	1	D. reticulatus	manual collection	dog	Unrecorded	19°C		
28-09-17	Livno	Livno	1	0	1	D. reticulatus	manual collection	horse	Unrecorded	22°C		
09-04-17	Velika Kladuša	Velika Kladuša	1	0	1	D. reticulatus	manual collection	goat	Unrecorded	17°C		
10-04-17	Bosanska Krupa	Bosanska Krupa	1	0	1	D. reticulatus	dragging	vegetation	Unrecorded	15°C	meadow	
20-04-23	Sarajevo	Hadžići	1	1	2	D. reticulatus	manual collection	cat	Cloudy	Unrecorded		Central Bosnia
20-04-23	Sarajevo	Ilidža	2	1	3	D. reticulatus	manual collection	dog	Sunny	Unrecorded		
21-04-23	Sarajevo	Ilijaš	2	1	3	D. reticulatus	manual collection	dog	Sunny	Unrecorded		
23-04-23	Sarajevo	Novo Sarajevo	3	1	4	D. reticulatus	manual collection	dog	Cloudy	Unrecorded	meadow	
23-04-23	Sarajevo	Novi Grad	2	1	3	D. reticulatus	manual collection	dog	Cloudy	Unrecorded		
24-04-23	Sarajevo	Vogošća	2	1	3	D. reticulatus	manual collection	dog	Sunny	Unrecorded		
27-04-23	Breza	Breza	3	1	4	D. reticulatus	manual collection	dog	Sunny	Unrecorded		
13-05-23	Bugojno	Bugojno	3	1	4	D. reticulatus	manual collection	dog	Sunny	13°C		
19-09-22	Vareš	Vareš	2	0	2	D. reticulatus	manual collection	cat	Sunny	Unrecorded		
27-05-22	Zenica	Zenica	2	0	2	D. reticulatus	manual collection	dog	Sunny	Unrecorded		
27-05-22	Visoko	Visoko	2	0	2	D. reticulatus	manual collection	dog	Sunny	Unrecorded		
07-04-22	Pale	Pale	2	0	2	D. reticulatus	manual collection	dog	Sunny	Unrecorded		
16-03-22	Bugojno	Bugojno	3	3	6	D. reticulatus	manual collection	dog	Sunny	Unrecorded		
15-03-22	Trnovo	Trnovo	4	0	4	D. reticulatus	manual collection	dog	Cloudy	12°C		

02-04-21	Bugojno	Bugojno	3	0	3	D. reticulatus	manual collection	dog	Cloudy	18°C	
10-05-21	Sarajevo	Novo Sarajevo	5	2	7	D. reticulatus	manual collection	dog	Sunny	17°C	
14-06-21	Sarajevo	Novi Grad	3	1	4	D. reticulatus	manual collection	dog	Sunny	18°C	
14-06-21	Sarajevo	Stari Grad	2	1	3	D. reticulatus	manual collection	cat	Sunny	18°C	
10-05-21	Trnovo	Trnovo	1	0	1	D. reticulatus	manual collection	cat	Sunny	15°C	
22-04-21	Sarajevo	Hadžići	1	0	1	D. reticulatus	manual collection	dog	Sunny	12°C	
22-04-21	Sarajevo	Ilidža	3	0	3	D. reticulatus	manual collection	dog	Sunny	12°C	
22-04-21	Sarajevo	Ilijaš	5	1	6	D. reticulatus	manual collection	dog	Sunny	12°C	
24-04-21	Sarajevo	Vogošća	3	0	3	D. reticulatus	manual collection	dog	Sunny	12°C	
23-04-21	Sarajevo	Centar	1	0	1	D. reticulatus	manual collection	dog	Cloudy	12°C	
09-05-21	Zenica	Zenica	2	0	2	D. reticulatus	manual collection	dog	Sunny	14°C	
09-05-21	Visoko	Visoko	1	0	1	D. reticulatus	manual collection	dog	Sunny	14°C	
04-07-21	Breza	Breza	1	1	2	D. reticulatus	manual collection	dog	Sunny	21°C	
10-10-21	Sarajevo	Ilidža	3	2	5	D. reticulatus	manual collection	dog	Sunny	19°C	
28-03-20	Sarajevo	Ilijaš	1	0	1	D. reticulatus	manual collection	dog	Sunny	Unrecorded	
22-03-20	Sarajevo	Novo Sarajevo	3	1	4	D. reticulatus	manual collection	dog	Sunny	Unrecorded	
22-03-20	Sarajevo	Novi Grad	1	1	2	D. reticulatus	manual collection	dog	Sunny	Unrecorded	
22-03-20	Sarajevo	Vogošća	2	0	2	D. reticulatus	manual collection	dog	Sunny	Unrecorded	
22-03-20	Trnovo	Trnovo	2	1	3	D. reticulatus	manual collection	dog	Sunny	Unrecorded	

19-03-20	Visoko	Visoko	2	2	4	D. reticulatus	manual collection	dog	Sunny	Unrecorded		
26-03-19	Sarajevo	Centar	1	2	3	D. reticulatus	manual collection	dog	Sunny	Unrecorded		
27-03-19	Sarajevo	Stari Grad	1	0	1	D. reticulatus	manual collection	dog	Sunny	Unrecorded		
28-03-19	Trnovo	Trnovo	3	2	5	D. reticulatus	manual collection	dog	Sunny	Unrecorded		
11-04-19	Sarajevo	Nahorevo	2	1	3	D. reticulatus	manual collection	dog	Cloudy	Unrecorded		
11-04-19	Sarajevo	Novi Grad	1	1	2	D. reticulatus	manual collection	dog	Sunny	Unrecorded		
12-04-19	Sarajevo	Centar	2	1	3	D. reticulatus	manual collection	dog	Sunny	Unrecorded		
15-04-19	Sarajevo	Bjelašnica	2	0	2	D. reticulatus	manual collection	dog	Sunny	Unrecorded		
17-04-19	Sarajevo	Hadžići	6	2	8	D. reticulatus	manual collection	dog	Sunny	Unrecorded		
16-05-19	Sarajevo	Ilidža	6	1	7	D. reticulatus	manual collection	dog	Sunny	Unrecorded		
28-05-18	Zenica	Zenica	3	1	4	D. reticulatus	manual collection	cat	Cloudy	Unrecorded		
24-10-18	Sarajevo	Otoka	3	1	4	D. reticulatus	manual collection	dog	Sunny	19°C		
05-05-17	Sarajevo	Novo Sarajevo	0	1	1	D. reticulatus	manual collection	dog	Sunny	17°C		
12-05-17	Sarajevo	Novi Grad	1	0	1	D. reticulatus	manual collection	dog	Sunny	21°C		
20-04-17	Sarajevo	Vogošća	1	1	2	D. reticulatus	manual collection	dog	Sunny	18°C		
22-03-17	Trnovo	Trnovo	3	2	5	D. reticulatus	manual collection	dog	Sunny	13°C		
18-02-23	Mostar	Mostar	2	1	3	D. reticulatus	manual collection	dog	Sunny	8°C		Herzegovina
22-02-23	Mostar	Rodoč	2	0	2	D. reticulatus	manual collection	dog	Sunny	10°C		
09-03-23	Stolac	Stolac	1	0	1	D. reticulatus	manual collection	dog	Sunny	10°C		

14-04-23	Jablanica	Jablanica	1	1	2	D. reticulatus	manual collection	dog	Sunny	13°C		
14-04-23	Konjic	Konjic	1	0	1	D. reticulatus	manual collection	dog	Sunny	13°C		
22-04-23	Berkovići	Berkovići	1	0	1	D. reticulatus	manual collection	dog	Sunny	Unrecorded		
22-04-23	Nevesinje	Nevesinje	2	0	2	D. reticulatus	manual collection	dog	Sunny	Unrecorded		
22-04-23	Bileća	Bileća	0	2	2	D. reticulatus	manual collection	sheep	Sunny	Unrecorded		
23-04-23	Čitluk	Čitluk	2	0	2	D. reticulatus	manual collection	dog	Sunny	Unrecorded		
23-04-23	Međugorje	Međugorje	6	1	7	D. reticulatus	dragging	vegetation	Sunny	Unrecorded	meadow	
23-04-23	Međugorje	Međugorje	5	1	6	D. reticulatus	manual collection	dog	Sunny	Unrecorded		
23-04-23	Ljubuški	Ljubuški	1	0	1	D. reticulatus	manual collection	dog	Sunny	Unrecorded		
18-04-23	Gacko	Gacko	1	0	1	D. reticulatus	manual collection	dog	Sunny	15°C		
08-06-23	Čapljina	Čapljina	1	0	1	D. reticulatus	manual collection	dog	Sunny	20°C		
01-09-23	Grude	Grude	2	0	2	D. reticulatus	manual collection	dog	Sunny	21°C		
03-09-22	Mostar	Mostar	1	0	1	D. reticulatus	manual collection	dog	Sunny	23°C		
03-09-22	Međugorje	Međugorje	3	0	3	D. reticulatus	manual collection	dog	Sunny	23°C		
12-09-22	Jablanica	Jablanica	2	2	4	D. reticulatus	manual collection	dog	Sunny	19°C		
16-06-22	Čapljina	Čapljina	1	0	1	D. reticulatus	manual collection	dog	Sunny	21°C		
27-05-22	Nevesinje	Nevesinje	2	0	2	D. reticulatus	manual collection	cattle	Sunny	18°C		
07-04-22	Mostar	Mostar	3	0	3	D. reticulatus	manual collection	dog	Sunny	11°C		
16-03-22	Mostar	Mostar	2	0	2	D. reticulatus	manual collection	dog	Sunny	13°C		

15-03-22	Jablanica	Jablanica	4	0	4	D. reticulatus	manual collection	dog	Sunny	12°C	
10-02-21	Čapljina	Čapljina	1	0	1	D. reticulatus	manual collection	dog	Sunny	Unrecorded	
21-03-21	Međugorje	Međugorje	6	0	6	D. reticulatus	manual collection	dog	Sunny	15°C	
21-03-21	Stolac	Stolac	2	0	2	D. reticulatus	manual collection	cattle	Sunny	15°C	
06-02-21	Čitluk	Čitluk	2	0	2	D. reticulatus	manual collection	dog	Sunny	Unrecorded	
06-02-21	Ljubuški	Ljubuški	2	0	2	D. reticulatus	manual collection	dog	Sunny	Unrecorded	
21-09-21	Mostar	Rodoč	3	0	3	D. reticulatus	manual collection	dog	Sunny	17°C	
22-09-21	Mostar	Buna	1	0	1	D. reticulatus	dragging	vegetation	Sunny	17°C	meadow
22-09-21	Čapljina	Čapljina	1	0	1	D. reticulatus	manual collection	dog	Sunny	17°C	
14-10-21	Jablanica	Jablanica	1	1	2	D. reticulatus	manual collection	dog	Sunny	17°C	
28-03-20	Stolac	Stolac	1	1	2	D. reticulatus	manual collection	dog	Sunny	14°C	
25-03-20	Mostar	Mostar	1	0	1	D. reticulatus	manual collection	dog	Sunny	16°C	
25-03-20	Jablanica	Jablanica	4	1	5	D. reticulatus	manual collection	dog	Sunny	15°C	
25-03-20	Konjic	Konjic	3	0	3	D. reticulatus	manual collection	dog	Sunny	16°C	
25-03-20	Mostar	Buna	2	0	2	D. reticulatus	manual collection	cat	Sunny	17°C	
25-03-20	Mostar	Mostar	2	0	2	D. reticulatus	manual collection	dog	Sunny	17°C	
25-03-20	Čapljina	Čapljina	1	0	1	D. reticulatus	manual collection	dog	Sunny	17°C	
20-09-20	Jablanica	Jablanica	1	1	2	D. reticulatus	manual collection	dog	Sunny	14°C	
07-05-19	Konjic	Konjic	2	0	2	D. reticulatus	manual collection	dog	Sunny	15°C	

28-05-19	Stolac	Stolac	1	0	1	D. reticulatus	manual collection	dog	Sunny	17°C		
19-03-18	Jablanica	Jablanica	3	0	3	D. reticulatus	manual collection	dog	Sunny	11°C		
08-04-18	Čapljina	Čapljina	1	0	1	D. reticulatus	manual collection	dog	Sunny	14°C		
18-07-18	Berkovići	Berkovići	1	0	1	D. reticulatus	manual collection	dog	Sunny	20°C		
17-09-23	Bileća	Bileća	2	0	2	D. reticulatus	dragging	vegetation	Sunny	19°C	underbrush	
18-07-18	Bileća	Bileća	2	0	2	D. reticulatus	manual collection	dog	Sunny	20°C		
19-09-18	Ljubuški	Ljubuški	1	0	1	D. reticulatus	manual collection	dog	Sunny	18°C		
17-06-18	Grude	Grude	3	0	3	D. reticulatus	manual collection	horse	Sunny	18°C		
18-09-17	Stolac	Stolac	2	0	2	D. reticulatus	manual collection	goat	Sunny	17°C		
10-06-17	Konjic	Konjic	1	0	1	D. reticulatus	manual collection	dog	Sunny	15°C		
10-06-17	Jablanica	Jablanica	3	0	3	D. reticulatus	manual collection	dog	Sunny	14°C		
10-06-17	Čitluk	Čitluk	1	1	2	D. reticulatus	manual collection	dog	Sunny	16°C		
10-06-17	Ljubuški	Ljubuški	1	0	1	D. reticulatus	manual collection	dog	Sunny	16°C		
23-04-17	Mostar	Mostar	1	0	1	D. reticulatus	manual collection	dog	Sunny	15°C		
07-04-22	Foča	Tjentište	1	0	1	D. reticulatus	manual collection	dog	Sunny	13°C		
16-03-22	Goražde	Goražde	1	0	1	D. reticulatus	manual collection	dog	Sunny	13°C		
15-09-22	Goražde	Goražde	1	0	1	D. reticulatus	manual collection	dog	Sunny	16°C		E Bosnia
02-03-21	Rudo	Rudo	1	0	1	D. reticulatus	manual collection	dog	Sunny	9°C		
28-03-20	Rogatica	Rogatica	1	0	1	D. reticulatus	manual collection	dog	Sunny	15°C		

22-03-20	Goražde	Goražde	0	1	1	D. reticulatus	manual collection	dog	Sunny	13-14°C		
16-05-19	Srebrenica	Srebrenica	1	0	1	D. reticulatus	manual collection	dog	Sunny	15°C		
24-05-23	Banja Luka	Banja Luka	1	1	2	D. reticulatus	manual collection	dog	Sunny	13°C		
21-06-23	Tuzla	Tuzla	2	2	4	D. reticulatus	manual collection	dog	Sunny	17°C		
21-06-23	Srebrenik	Srebrenik	5	0	5	D. reticulatus	manual collection	dog	Sunny	17°C		
25-03-22	Banja Luka	Banja Luka	1	0	1	D. reticulatus	manual collection	dog	Sunny	14°C		
25-03-22	Laktaši	Laktaši	1	0	1	D. reticulatus	manual collection	dog	Sunny	13-15°C		
25-03-22	Ključ	Ključ	1	0	1	D. reticulatus	manual collection	dog	Sunny	Unrecorded		
12-09-22	Tešanj	Tešanj	1	0	1	D. reticulatus	manual collection	dog	Sunny	Unrecorded		
13-09-22	Banja Luka	Banja Luka	1	0	1	D. reticulatus	manual collection	dog	Sunny	Unrecorded		
22-09-22	Laktaši	Laktaši	1	2	3	D. reticulatus	manual collection	dog	Sunny	Unrecorded		N and NE Bosnia
10-05-21	Ključ	Ključ	4	3	7	D. reticulatus	manual collection	dog	Sunny	14°C		
10-05-21	Banja Luka	Banja Luka	1	0	1	D. reticulatus	manual collection	dog	Sunny	16°C		
12-09-20	Laktaši	Laktaši	1	0	1	D. reticulatus	manual collection	dog	Sunny	Unrecorded		
19-04-20	Tuzla	Tuzla	2	2	4	D. reticulatus	manual collection	dog	Sunny	15°C		
21-05-20	Laktaši	Laktaši	2	0	2	D. reticulatus	manual collection	dog	Sunny	Unrecorded		
21-05-20	Ključ	Ključ	1	0	1	D. reticulatus	manual collection	dog	Sunny	Unrecorded		
28-05-19	Živinice	Živinice	1	0	1	D. reticulatus	manual collection	dog	Sunny	16°C		
19-03-18	Tuzla	Tuzla	5	1	6	D. reticulatus	manual collection	dog	Sunny	Unrecorded		

08-04-18	Gračanica	Gračanica	1	0	1	D. reticulatus	manual collection	dog	Sunny	Unrecorded		
15-07-18	Srebrenik	Srebrenik	1	1	2	D. reticulatus	manual collection	dog	Sunny	16-18°C		
15-07-18	Lukavac	Lukavac	1	1	2	D. reticulatus	manual collection	dog	Sunny	16-18°C		
15-07-18	Tuzla	Tuzla	1	1	2	D. reticulatus	manual collection	dog	Sunny	16-18°C		
